# The effect of food ration bar enriched with β‐alanine, L‐arginine, and Nigella sativa on performance and inflammation following intense military training: A double‐blind randomized clinical trial

**DOI:** 10.1002/fsn3.2297

**Published:** 2021-05-06

**Authors:** Saeid Hadi, Mahsa Miryan, Davood Soleimani, Reza Amani, Mostafa Mazaheri Tehrani, Vahid Hadi, Morad Esmaiil Zali, Negin Mosalmanzadeh, Gholamreza Askari

**Affiliations:** ^1^ Department of Community Nutrition School of Nutrition and Food Sciences Isfahan University of Medical Sciences Isfahan Iran; ^2^ Department of Health, Science and Research Branch AJA University of Medical Sciences Tehran Iran; ^3^ Nutrition Research Center Student Research Committee Department of Clinical Nutrition School of Nutrition and Food Sciences Tabriz University of Medical Sciences Tabriz Iran; ^4^ Nutritional Sciences Department School of Nutrition Sciences and Food Technology Kermanshah University of Medical Sciences Kermanshah Iran; ^5^ Department of Food Science and Technology Faculty of Agriculture Ferdowsi University of Mashhad Mashhad Iran; ^6^ Department of Management and Health Economy Faculty of Medicine AJA University of medical sciences Tehran Iran; ^7^ Department of Nutrition Sciences Varastegan Institute for Medical Sciences Mashhad Iran; ^8^ Food Security Research Center Isfahan University of Medical Sciences Isfahan Iran

**Keywords:** inflammation, L‐arginine, military training, Nigella sativa, VO_2_ max, β‐alanine

## Abstract

**Background:**

There are growing interests in using dietary supplements to improve athletic performance. This study aimed to evaluate the effect of the food ration bar enriched with β‐alanine, L‐arginine, and Nigella sativa on athletic performance and inflammation following intense military training.

**Methods:**

This double‐blind, randomized, controlled clinical trial was conducted on 54 new cadets. Eligible participants were randomly assigned in a 1:1 ratio to receive food ration bars enriched with arginine (2 g/day), β‐alanine (2 g/day), and Nigella sativa (2 g/day) or nonenriched food ration bars during a 2‐week military training. Aerobic and anaerobic performances were evaluated by the Cooper and RAST tests, respectively.

**Results:**

A significant increase in anaerobic powers (min, mean, and max) and a significant reduction in fatigue index were observed in the intervention group as compared to the control group, even after the adjustment for confounding factors. Also, increased levels of hs‐CRP and TNF‐α following military training were significantly lower in the intervention group as compared to the control group (hs‐CRP: 0.55 ± 0.1 versus 2.43 ± 0.1 mg/L; *p*‐value: 0.01; TNF‐α: 0.12 ± 0.04 versus 0.62 ± 0.04 pg/ml; *p*‐value: 0.03). No significant changes were observed in VO_2_ max in both groups.

**Conclusions:**

Our results showed that the combination of β‐alanine, L‐arginine, and Nigella sativa can improve anaerobic performance and reduce inflammation following intense physical activities. Further studies with long‐term duration are needed to confirm the cumulative/synergic effects of these ingredients in trained and nontrained subjects.

## Graphical abstract

This clinical trial study shows that the combination of β‐alanine, L‐arginine, and Nigella sativa can improve anaerobic performance and reduce inflammation following intense physical activities. Further studies with long‐term duration are needed to confirm the cumulative/synergic effects of these ingredients in trained and non‐trained subjects. As far as we know, this is the first trial to evaluate the effect of the combination of β‐alanine, L‐arginine, and Nigella sativa on exercise performance.

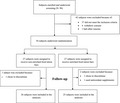

## 1 INTRODUCTION

Nutrition status plays an important role in the mental and physical performance of military personnel in combat situations (Hill et al., 2011). In recent years, the use of dietary supplements among military personnel has been placed in the spotlight. It has been estimated that more than 55% of military personnel in the world use dietary supplements (Knapik et al., 2014). Military personnel usually use dietary supplements to increase performance rather than to compensate for nutritional deficiencies (Jovanov et al., 2019).

L‐arginine is a popular nutritional supplement among athletes that is claimed to enhance vasodilation and increase blood flow to active muscles by raising nitric oxide production (Maxwell et al., 2001). Increased blood flow in active muscle leads to higher delivery of substrates (e.g., oxygen and nutrients) to active muscles and speeds up the removal of the metabolic wastes, which can subsequently improve athletic performance and delay fatigue (Hadi et al., 2020; Joyner & Casey, 2015). Beta‐alanine is another popular supplement for athletes which acts as a carnosine precursor in skeletal muscles (Stellingwerff et al., 2012). Evidence revealed that intramuscular acidosis due to the accumulation of anaerobic glycolysis products, lactate and hydrogen ions, inhibits glycolysis pathway enzymes and results in fatigue in skeletal muscle under intense and short‐term activity (Cairns, 2006). Carnosine, with its tampon capacity, is declared to be a principal contributor in regulating the acid–base balance in skeletal muscle and improving athlete performance in strenuous exercises (Stellingwerff et al., 2012). Previous literature shows that beta‐alanine supplementation increases skeletal muscle carnosine content by 65%, followed by a 15–25% increase in their buffering capacity (Harris et al., 2006).

Intense military training increases the production of reactive oxygen species (ROS) (Tanskanen et al., 2011). The increase of ROS can cause irreversible changes in the structure of the biological macromolecules, impaired muscle contractile function, inflammation, and atrophy of skeletal muscles (Gomez‐Merino et al., 2003). Nigella sativa (*N*. *sativa*) has long been widely used for flavoring and medical purposes throughout the world (Botnick et al., 2012). Nigella sativa oil contains active compounds such as thymol, thymoquinone, and dithymoquinone. Thymoquinone is a direct scavenger of ROS and enhances the capacity of the body's antioxidant defense system through modulating the transcription factor Nrf1 (Hadi et al., 2016; Hu et al., 2019). In line with the above explanations, a study on rats showed that consumption of Nigella sativa reduces oxidative stress and inflammation following intense exercise (Gholamnezhad et al., 2014). Taken together, supplementation with a combination of β‐alanine, L‐arginine, and Nigella sativa seems to have favorable effects on performance in athletes and military personnel through different cellular mechanisms. Therefore, this trial aims to investigate the efficacy of the food ration bar enriched with β‐alanine, L‐arginine, and Nigella sativa on exercise performance and inflammation following intense military training.

## 2 METHODS AND MATERIALS

### 2.1 Study design and participants

The current study is a double‐blind, randomized, controlled clinical trial that examined the efficacy of the food ration bar enriched with β‐alanine, arginine, and Nigella sativa on exercise performance and inflammation following intense military training. The trial was conducted in accordance with the principles of the Helsinki Declaration after approval by the ethics committee of the Isfahan University of Medical Sciences, Isfahan, Iran. Moreover, the study protocol has been registered in the Iranian Registry of Clinical Trials (ID: IRCT20121216011763N43). In this study, sampling was based on the volunteer sampling method. Samples were recruited through word of mouth and poster installation in the dormitory of AJA University of Medical Sciences in Tehran, Iran. Written consent was then obtained from all participants after gaining knowledge of the objectives, process, and potential risks and benefits of the study.

Ninety‐six volunteers enrolled in the trial and were assessed for eligibility criteria. Volunteers were eligible for inclusion if they were men, age 20–30 years, had a body mass index (BMI) between 18.5 and 25 kg/m^2^, and were athletes. Exclusion criteria included any history of sports injuries, smoking, medical conditions including diabetes, hypertension, and metabolic disorders such as glycogen storage disorders, musculoskeletal disorders, regular use of sport and antioxidant supplements, anti‐inflammatory drugs, and the history of allergy to supplements containing Nigella sativa, β‐alanine, or arginine. During the study, participants who were reluctant to cooperate, adhered to less than 80% of their assigned intervention, used dietary supplements during the trial, or developed allergies were withdrawn from the study.

### 2.2 Trial randomization and intervention

After obtaining the eligibility criteria, 54 participants were randomly assigned to the intervention or control group in the ratio 1:1. Allocation sequences for assigning participants to the study groups were obtained by an independent statistician using a random‐number table. These sequences remained hidden in opaque, numbered, and sealed envelopes until the end of the eligibility criteria evaluation.

All participants took part in 300 min/day of a military training program in two sessions which lasted for 2 weeks. During this course, participants in the intervention group received two 100‐gram food ration bars enriched with 340 mg arginine, 340 mg β‐alanine, and 340 mg Nigella sativa oil three times a day. Participants in the control group received two 100 g of nonenriched food ration bars three times a day for 2 weeks. Each food ration bar contained 525 Kcal with 13% of calories as protein, 42% as fat, and 45% as carbohydrate. All the food ration bars were produced by the Department of Food Science and Technology at the Ferdowsi University of Mashhad in Iran under Manufacturing Practice conditions. Both food ration bars were quite similar in shape, color, smell, taste, weight, and packaging. In this study, participants' compliance was assessed by counting the remaining food ration bars at the end of each week. In this study, the assignment of participants to study groups was hidden from all participants and researchers until the end of the study and data analysis.

### 2.3 Assessment of exercise performance

In order to measure the participants' anaerobic performance, a running‐based anaerobic sprint test (RAST) was completed (Zagatto et al., 2009). After informing the study participants about the test protocols, they ran a distance of 35 m six times with 10‐s rest intervals. Given the final time obtained for each 35‐meter run, anaerobic performance indices including power (watts) and fatigue index (watts/s) were obtained through the following formulas:

$$\text{Power}\;:\;\left(\text{Weight}\;\times \;{\text{Distance}}^{2}\right)\;/\;{\text{Time}}^{3}$$

FatigueIndex:(PowerMax‐PowerMin)/Timespentinsixsprints

Participants' aerobic performance was assessed using the Cooper 12‐min run test (Cooper, 1968). After sufficient explanations of the test protocol, participants ran on a 400‐meter track for 12 min, and the distance traveled was recorded at the end of the time. According to the distance traveled in these 12 min, VO_2_ max as an indicator for aerobic capacity was obtained through the following formula:

$${\text{VO}}_{2}\;\text{max}\;=\;\left(\text{Recorded}\;\text{Distance}\;‐\;504.9\right)\;÷\;44.73$$

Before the start of each exercise test, participants warmed up for 10 min and, at the end of the test, actively cooled down for 5 min. Participants were also encouraged by spectators and researchers during the tests.

### 2.4 Biochemical assessment

Before and after 2 weeks of basic military training, blood samples were collected in heparinized tubes and immediately centrifuged. Afterward, plasma samples were used immediately to assess levels of tumor necrosis factor alpha (TNF‐α) and high‐sensitivity C‐reactive protein (hs‐CRP). The enzyme‐linked immunosorbent assay (ELISA) method based on the biotin double antibody sandwich technique was done to measure the plasma levels of TNF‐α using the commercial kit (Diaclone, Besancon Cedex). Plasma levels of hs‐CRP were measured based on the colorimetric method using the commercial kit (Pars Azmoun kit) with a biochemistry autoanalyzer (Alfa‐Classic; Tajhizat Sanjesh Co., Ltd.).

### 2.5 Statistical analysis

The statistical package for the social sciences (SPSS) software version 16 (SPSS Inc.) was used to analyze data. Each study group needed to be 27 participants to detect a difference of one standard deviation (*SD*) in the TNF‐α between the intervention and control group with an anticipated dropout rate of 20% and power 90% at a significance level of 5% (Darand et al., 2019). Within‐group differences were ascertained using paired‐sample *t* test and Wilcoxon rank‐sum test. Also, between‐group differences were ascertained using independent Student's *t* test and Mann–Whitney U test. Analysis of covariance (ANCOVA) was used to adjust baseline values and change in weight as covariates. A *p*‐value of less than 0.05 was considered to indicate statistical significance.

## 3 RESULTS

**FIGURE 1:**
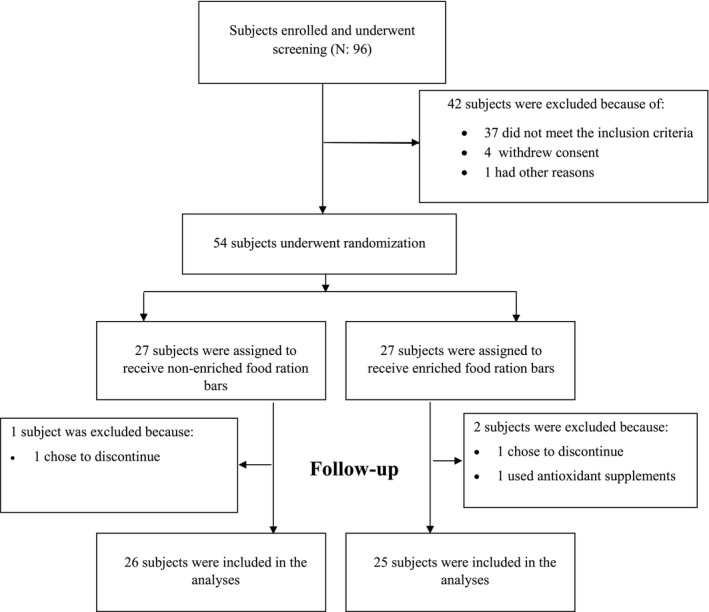
Screening, randomization, treatment, follow‐up

A total of 96 cadets were enrolled in the trial and were screened for eligibility criteria, 54 of whom underwent randomization and were randomly assigned to the intervention group (*N* = 27) or control group (*N* = 27). Two participants in the intervention group and one participant in the control group discontinued the trial for reasons unrelated to the study (Figure 1). There was no significant difference in the dropout rate between the intervention and control group (OR = 1.56; *p*‐value = .64). The mean compliance rate was 95.3% in the intervention group and 93.7% in the control group (*p*‐value = .81). No adverse events were reported in both groups throughout the trial.

The mean baseline age, weight, height, and BMI of participants were 23.55 ± 1.95 years, 74.46 ± 2.87 kg, 1.76 ± 0.03 m, and 23.79 ± 0.99 kg/m^2^, respectively. The baseline characteristics of participants in the intervention and placebo groups are shown in Table 1. There were no significant differences between the intervention and placebo groups in terms of baseline characteristics (*p*‐value > .05). At the end of the trial, the mean weight was 74.02 ± 2.74 kg in the intervention group and 74.51 ± 3.02 kg in the control group. The mean changes in the weight did not differ significantly between the intervention and control groups (−0.14 ± 0.49 and −0.23 ± 0.72 kg, respectively, *p*‐value: 0.58).

**TABLE 1:** Baseline characteristics of participants in the intervention and placebo groups

Variables	Intervention group (*N*:25)	Placebo group (*N*:26)	*p*‐Value*
Age, years	23.41 ± 2.02	23.69 ± 1.9	.59
Weight; kg	74.16 ± 2.86	74.75 ± 2.90	.47
Height; m	1.77 ± 0.037	1.76 ± 0.026	.23
Body mass index; kg/m^2^	23.55 ± 1.01	24.03 ± 0.93	.16

Note

Data are presented as mean ± standard deviation.

* *p*‐Values were obtained from the independent‐sample *t* test.

The adjusted mean changes in the anaerobic and aerobic exercise parameters from baseline to the end of the trial are shown in Table 2. The baseline values of power (min, mean, and max) and fatigue index in the RAST test and VO_2_ max in the Cooper's did not differ significantly between the two groups (*p*‐value > .05). At the end of the trial, the power (min, mean, and max) and VO_2_ max increased and fatigue index decreased significantly in the intervention group, while a significant increase in VO_2_ max and reduction in the power (min and mean) were observed in the control group. As shown in Table 2, the changes in power (min, mean, and max) and fatigue index remained significant in the intervention group as compared to the control group, even after the adjustment for baseline values and changes in weight.

**TABLE 2:** Adjusted mean changes in anaerobic and anaerobic performance from baseline to the end of the trial

Variables	Group	Before	After	*p*‐Value*	Mean changes**
Power Min; watts	Intervention	343.73 ± 50.51	363.33 ± 49.70	.006	16.16 ± 1.89
	Control	365.11 ± 45.91	332.51 ± 67.83	.001	−28.93 ± 1.81
	*p*‐Value***	0.120	0.059	^–^	0.002
Power Max; watts	Intervention	644.01 ± 88.94	694.35 ± 110.99	.010	44.06 ± 3.98
	Control	683.39 ± 133.57	663.62 ± 131.85	.221	−13.73 ± 3.82
	*p*‐Value***	0.223	0.373	^–^	0.045
Power Mean; watts	Intervention	463.97 ± 34.43	482.25 ± 43.07	.043	12.93 ± 1.53
	Control	482.24 ± 43.07	457.11 ± 41.32	.001	−19.99 ± 1.48
	*p*‐Value***	0.130	0.038	–	0.004
FI; watts/s	Intervention	9.59 ± 3.07	7.89 ± 2.86	.026	−1.63 ± 0.113
	Control	9.27 ± 4.42	9.67 ± 3.74	.522	0.34 ± 0.108
	*p*‐Value***	0.762	0.062	–	0.016
VO_2_ max; ml kg^−1^ min^−1^	Intervention	46.16 ± 2.78	49.68 ± 3.58	.001	3.56 ± 0.111
	Control	45.54 ± 1.82	48.35 ± 3.21	.001	2.78 ± 0.107
	*p*‐Value***	0.347	0.167	–	0.315

Note

Data are presented as mean ± standard deviation.

Abbreviation: FI, fatigue index.

* *p*‐Values were obtained from paired‐sample *t* test.

** Values were obtained from ANCOVA test with energy intake and physical activity as covariates.

*** *p*‐Values were obtained from the Independent‐sample *t* test.

The adjusted mean changes in the inflammatory parameters from baseline to the end of the trial are shown in Table 3. The baseline values of hs‐CRP and TNF‐α did not differ significantly between the two groups (*p*‐value > .05). But their levels were significantly lower in the intervention group compared to the control group. These observations remained significant even after the adjustment for baseline values and changes in weight.

**TABLE 3:** Adjusted mean changes in inflammatory parameters from baseline to the end of the trial

Characteristic	Group	Before	After	*p*‐Value*	Changes**
TNF‐α; pg/ml	Intervention	7.03 ± 2.42	7.55 ± 3.48	.44	0.557 ± 0.106
	Control	7.27 ± 2.35	9.73 ± 3.15	.001	2.432 ± 0.102
	*p*‐Value***	0.718	0.023	^–^	0.015
hs‐CRP; mg/l	Intervention	1.62 ± 0.76	1.71 ± 0.69	.373	0.12 ± 0.043
	Control	1.65 ± 0.65	2.29 ± 1.69	.034	0.62 ± 0.041
	*p*‐Value***	0.77	0.61	^–^	0.032

Note

Data are presented as mean ± standard deviation.

Abbreviations: hs‐CRP, high‐sensitivity C‐reactive protein; TNF‐α, tumor necrosis factor alpha.

* *p*‐Values were obtained from paired‐sample *t* test.

** Values were obtained from ANCOVA test with energy intake and physical activity as covariates.

*** *p*‐Values were obtained from the independent‐sample *t* test.

## 4 DISCUSSION

**FIGURE 2:**
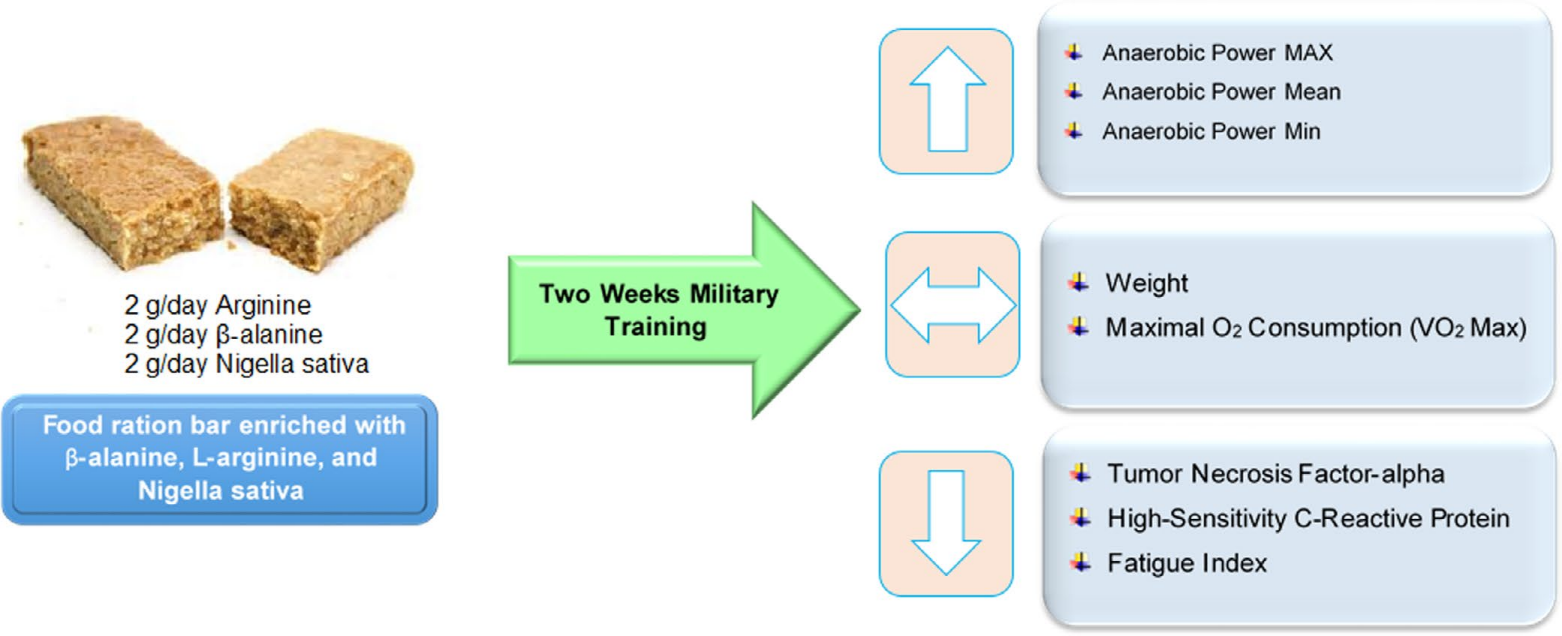
The effect of food ration bar enriched with β‐alanine, L‐arginine, and nigella sativa on performance and inflammation following intense military training. [Down arrow (decrease); up arrow (increase); double‐sided arrow (without change)]

To our knowledge, the current clinical trial is the first report of the effects of a combination of β‐alanine, L‐arginine, and Nigella sativa on aerobic and anaerobic performance and inflammation following intense military training. Our results show that the food ration bar enriched with a combination of β‐alanine, L‐arginine, and Nigella sativa improved power and FI during an anaerobic test, while it had no significant effect on VO_2_ max during the Cooper aerobic test. In addition to sports performance, it significantly reduced the serum levels of TNF‐α and hs‐CRP following intense military training (Figure 2).

Our results show that supplementation with a combination of β‐alanine, L‐arginine, and Nigella sativa had no effect on the body's aerobic capacity predicted by VO_2_ max. L‐arginine is one of the most common ingredients in many dietary supplements claimed to improve aerobic performance due to its role in the synthesis of nitric oxide and subsequently increasing blood flow and nutrients and oxygen supply to skeletal muscles (Maxwell et al., 2001). However, scientific evidence on the efficacy of L‐arginine supplementation on athletic performance is controversial and challenging. In a 4‐week randomized clinical trial, Alvares et al. found that the daily administration of 6 g of encapsulated L‐arginine for 4 weeks had no significant effect on a 5‐km time‐trial running test in trained runners (Alvares et al., 2014). Likewise, Bailey et al. found that the daily intake of 6 g of L‐arginine over a period of 1 week did not affect the time to exhaustion during moderate‐intensity cycle exercise in active males (Bailey et al., 2015). Conversely, Pahlavani et al. found that the daily intake of 2 g of L‐arginine for 6 weeks improved VO_2_ max predicted by the Harvard step test among male soccer players (Pahlavani et al., 2017). Likewise, Camic et al. reported that the daily intake of 1.5 and 3 g of L‐arginine for 4 weeks improved physical working capacity during the incremental test to exhaustion among active men (Camic et al., 2010). These controversies are mainly due to the heterogeneity of the study population, dosage and length of use, and outcomes. In a recent systematic review, the authors concluded that 1.5–2 g of L‐arginine over a period of 4–7 weeks is needed to observe the improvement in aerobic performance (Viribay et al., 2020). Therefore, it seems likely that our findings in this line resulted from the short duration of use (2 weeks) and the absence of the cumulative/synergic effect between L‐arginine and other active ingredients.

We also found that supplementation with a combination of β‐alanine, L‐arginine, and Nigella sativa led to enhance the body's anaerobic capacity predicted by power and fatigue index. Supplementation with 1.6 g β‐alanine over a period of 2 weeks effectively increases muscle carnosine content (Stellingwerff et al., 2012), while the oral administration of carnosine does not influence muscle carnosine because of its breakdown in the gastrointestinal tract. Carnosine has been claimed to improve the anaerobic performance due to its buffering capacity in attenuation of exercise‐induced acidosis. In a 4‐week randomized clinical trial, Bellinger et al. found that β‐alanine supplementation at a daily dose of 6.4 g only improved anaerobic capacity by 5.5%, whereas it did not affect aerobic capacity in trained cyclists (Bellinger & Minahan, 2016). In another clinical trial, the administration of 6.4 g of β‐alanine for 4 weeks significantly improved the Wingate anaerobic power test in both trained and nontrained individuals (de Salles Painelli et al., 2014). Likewise, the daily intake of 2.4 g of β‐alanine for 4 weeks resulted in a significant improvement in mean power during the Wingate anaerobic test among female footballers (Rodríguez Rodríguez et al., 2014). Otherwise, a 3‐week clinical trial on female footballers showed that the administration of 6.4 g of β‐alanine had no effect on sprint times during the RAST (Ribeiro et al., 2020). Likewise, in another study, no significant improvement in Wingate anaerobic power test and fatigue rate in 200‐yd shuttle runs was observed with supplementation of 4.5 g β‐alanine during 4 weeks in football players (Hoffman et al., 2008). These controversies can be related to the heterogeneity of dosage and length of use. However, a recent systematic review and meta‐analysis revealed that β‐alanine supplementation at a daily dose of 3.2–6.4 g for 2–4 weeks is needed to observe the improvement in anaerobic performance (Saunders et al., 2020). Since we supplemented 2 g of β‐alanine for 2 weeks, the cumulative/synergic effects between β‐alanine and other active ingredients, especially L‐arginine, played an important role in our findings on anaerobic performance. In this line, L‐arginine is a precursor for the synthesis of creatine which acts as an important energy/pH buffer and supplies sufficient ATP for skeletal muscles at the beginning of intense activates for a few seconds (Barcelos et al., 2016). Consistent with this hypothesis, in a 4‐week clinical trial, Okudan et al. found that a combination of 1.6 g β‐alanine and 5 g creatine significantly improved mean power during Wingate anaerobic test than β‐alanine or creatinine alone among sedentary men (Okudan et al., 2015). Moreover, recent evidence shows that acute supplementation of L‐arginine at a dose of 0.15 g/kg at 60–90 min before exercise may improve anaerobic capacity due to speed up the removal of metabolic waste substances related to muscle fatigue, such as lactate and ammonia (Viribay et al., 2020).

Our results show that supplementation with a combination of β‐alanine, L‐arginine, and Nigella sativa reduced TNF‐α and h‐CRP following military training. Military training increased the levels of pro‐inflammatory mediators such as IL‐6 and TNF‐α which have a detrimental impact on muscle functions and immune responses to infection (Gomez‐Merino et al., 2003). Nigella sativa oil extract contains a considerable amount of aromatic organic compounds with anti‐inflammatory and anti‐oxidative properties such as thymoquinone, dihydrothymoquinon, p‐cymene, carvacrol, and thymol. In a study on treadmill‐exercised rats, the administration of Nigella sativa significantly reduced TNF‐α concentrations and TNFα/IL‐10 and IL‐6/IL‐10 ratio and increased IL‐10 (anti‐inflammatory cytokine) concentrations immediately after intense training (Gholamnezhad et al., 2014). Recent systematic review and meta‐analysis revealed that supplementation with Nigella sativa (1–3 g) reduced hs‐CRP by 0.5 mg/L among subjects with an inflammatory condition (Tavakoly et al., 2019). Nigella sativa has been shown to inhibit the activation of NF‐KB, a crucial transcription factor for the inflammatory gene expression, and the degradation of I‐KB as an inhibitor of the NF‐KB (Chehl et al., 2009). Recent systematic review and meta‐analysis revealed that L‐arginine supplementation did not affect CRP, IL‐6, and TNF‐α (Nazarian et al., 2019). However, there is evidence suggesting that L‐arginine can reduce inflammatory mediators (TNF‐α, IL‐1β, and IL‐6) and enhance muscle regeneration in Mdx mouse muscles through down‐regulation of the NF‐KB gene expression and inhibition of NF‐KB/metalloproteinase cascade (Hnia et al., 2008). In addition to L‐arginine, a randomized trial revealed the administration of 12 g/day β‐alanine for 7 days can increase IL‐10 concentrations during intense military training (Hoffman et al., 2018). Regarding inflammation, it can be concluded the anti‐inflammatory properties of our enriched ration bars can be due to the individual or cumulative effects of its active components.

The current trial was limited by the short duration of study because of the nature of our intervention (ration instead of routine diet) and the lack of a muscle biopsy (the measurement of muscle carnosine content) because of ethical consideration of using invasive procedures. Also, it is important to note that we cannot discriminate the effects of each agent from the others, since the three agents were consumed in combination, under our hypothesis that they might have favorable effects by complementary mechanisms. Nonetheless, our study has been strengthened with a minimal dropout rate, homogeneity of the study population, the similarity in terms of nutrients and energy among individuals during the intervention, adjustment for covariates, considerable similarity between two groups in baseline values, and high compliance rates.

In conclusion, our findings demonstrate that supplementation with a combination of β‐alanine, L‐arginine, and Nigella sativa might have beneficial effects on anaerobic performance and exercise‐induced inflammation in healthy active subjects. Further studies with long‐term duration (4–7 weeks) are needed to confirm the cumulative/synergic effects of β‐alanine, L‐arginine, and Nigella sativa on exercise performance in trained and nontrained subjects.

## CONFLICT OF INTEREST

All authors have declared no conflict of interest.

## AUTHOR CONTRIBUTIONS

**Saeid Hadi:** Conceptualization (equal); Data curation (equal); Investigation (equal); Methodology (equal); Project administration (equal); Validation (equal); Visualization (equal); Writing‐original draft (equal). **Mahsa Miryan:** Writing‐review & editing (equal). **Davood Soleimani:** Formal analysis (equal); Methodology (equal); Software (equal); Validation (equal); Writing‐review & editing (equal). **Reza Amani:** Methodology (equal); Writing‐original draft (equal). **Mostafa** **Mazaheri Tehrani:** Investigation (equal); Methodology (equal); Project administration (equal). **Vahid Hadi:** Data curation (equal); Investigation (equal). **Morad Esmaiil‐Zali:** Data curation (equal); Project administration (equal). **Negin Mosalmanzadeh:** Writing‐review & editing (equal). **Gholamreza Askari:** Conceptualization (equal); Data curation (equal); Formal analysis (equal); Funding acquisition (equal); Investigation (equal); Methodology (equal); Project administration (equal); Resources (equal); Software (equal); Supervision (equal); Validation (equal); Visualization (equal); Writing‐review & editing (equal).

## ETHICAL APPROVAL

The trial protocol was reviewed and approved in 8 March 2019 by the research ethics committee at the Isfahan University of Medical Sciences in Isfahan, Iran, following the Declaration of Helsinki (IR.MUI.RESEARCH.REC.1398.473). The trial was registered at the Iranian Registry of Clinical trial (number: IRCT20121216011763N43). All patients provided written consent for participation in this study.

## Data Availability

The data that support the findings of this study are available on request from the corresponding author. The data are not publicly available due to privacy or ethical restrictions.
